# Supplementary material for: Epidemiological Characteristics of Cancer Patients Attending at Felege Hiwot Referral Hospital, Northwest Ethiopia
Source: Int J Environ Res Public Health. 2023 Mar 22;20(6):5218. doi: 10.3390/ijerph20065218 (PMC10049658; doi:10.3390/ijerph20065218)
Supplement: Supplementary file 1 [file ijerph-20-05218-s001.zip › Supplementary data S1.pdf]

Health Facility Cancer Register

SN	MRN	Patient Name	Sex	Age	Address(region, zone,W,Tel #)	Assessed Risk factors (enter code)	Diagnosis with stage: Diagnosis	New (N)	Relapse/ Recurrence (RR)	Relapse/ on follow up(RF)	Pathology/lab results	Co-morbidity	Treatment Start Date	Type of treatment (Surgery, RT(Y/No), Targeted Therapy, Palliative care, chemo-Cycle	Supportive treatment (Mention all treatements given)	Outcome(I/C/D)
1	934227	[redacted]	M	46	Address: Bidar<br>Tel. 0918716875	f	CRC<br>Coro rectal ca			✓	CRC	No	12/9/14	Cycle 1: ✓<br>Cycle 2: ✓<br>Cycle 3: ✓<br>Cycle 4: ✓<br>Cycle 5: ✓<br>Cycle 6: ✓<br>Cycle 7:<br>Cycle 8:		
2	703003	[redacted]	M	62	Address: Aleta<br>Tel. 0924252586	f	Breast ca			✓	Breast ca	No	12/9/14	Cycle 1: ✓<br>Cycle 2: ✓<br>Cycle 3: ✓<br>Cycle 4: ✓<br>Cycle 5:<br>Cycle 6:<br>Cycle 7:<br>Cycle 8:		
3	902533	[redacted]	F	47	Address: Bidar<br>Tel. 0918728071	f	Breast		✓	✓	Breast ca		8/9/14	Cycle 1: ✓<br>Cycle 2: ✓<br>Cycle 3: ✓<br>Cycle 4: ✓<br>Cycle 5: ✓<br>Cycle 6: ✓<br>Cycle 7:<br>Cycle 8:		
4	927935	[redacted]	[redacted]	50	Address: Fofera<br>Tel. 0946230401<br>0928458981	f	Cervical ca			✓	Cervical ca		2/9/14	Cycle 1: ✓<br>Cycle 2: ✓<br>Cycle 3: ✓<br>Cycle 4: ✓<br>Cycle 5: ✓<br>Cycle 6: ✓<br>Cycle 7:<br>Cycle 8:		

Key for outcomes:- I= Improved, C= Cured, D= Death

New=N, Relapse/Recurrence=RR, Relapse/On fo[illegible]

a) Smoking b) unhealthy diet c) harmful use of alcohol d) physical inactivity e) Obesity f) others (mention)
