# Supplementary material for: Epidemiological Characteristics of Cancer Patients Attending at Felege Hiwot Referral Hospital, Northwest Ethiopia
Source: Int J Environ Res Public Health. 2023 Mar 22;20(6):5218. doi: 10.3390/ijerph20065218 (PMC10049658; doi:10.3390/ijerph20065218)
Supplement: Supplementary file 1 [file ijerph-20-05218-s001.zip › Supplementary data S2.pdf]

Bahir Dar University
College of Medicine and Health Sciences
ባህር ዳር ዩኒቨርስቲ ህክምናና ጤና ሳይንስ ኮሌጅ
Institutional Review Board

Wisdom at the Source of the Blue Nile
http://www.bdu.edu.et

ANNEX 3
Form CMHS/IRB 03-008

IRB's Decision

Meeting No.: 007/2018. Datejune6/6/ 2019
Protocol number: - 02066/18-09.................. Assigned No: 2018

Protocol Title:- *Epidemilogical Characterics Ofcancer patients attending at Felege Hiwot Referral Hospital, Amahara region,Northwest Ethiopia*	
Principal Investigator:	**Dr Muluken Azage**
Institute:	***College of Medicine and Health Sciences, Bahir Dar University***
Elements Reviewed (CMHS/IRB 01 - 008) :	☑ **Attached** ☐ Not attached
Review of Revised Application ☑ **Yes** ☐ No	Date of Previous review:
Decision of the meeting:	☑ **Approved** ☐ Approved with Recommendation ☐ Resubmission ☐ Disapproved

I. Elements approved:
1. Protocol Version No.: 01
2. Protocol Version Date 18/3/2019
3. Informed Consent Version: 01
4. Informed Consent Version Date: 18/3/2019

II. Obligations of the PI:
1. Comply with Standard National and International Ethical Guidelines
2. All Amendments and Changes made in the Protocol and Consent Needs IRB Approval
3. Report SAE within 10 days of the event
4. End of the study, including manuscript and thesis works should be reported to IRB

III. To NRERC ☐

Institutional Review Board (IRB) Approval: Period from…18/*03/2019* to 17/*03/2020*

Follow-up Report Expected in:
3months_______ 6months ✓_____ 9months__________ One Year__________

Chairperson, IRB
Dr Netsanet Fentahun
Signature__________
Date:..........................06/06/2019

ባህክምናና ጤና ሳይንስ ኮሌጅ ምርምር ተቋማዊ ቦርድ
College of Medicine and Health Sciences Institutional Review Board
